# Normal Development and Function of T Cells in Proline Rich 7 (Prr7) Deficient Mice

**DOI:** 10.1371/journal.pone.0162863

**Published:** 2016-09-22

**Authors:** Matous Hrdinka, Kritika Sudan, Sissy Just, Ales Drobek, Ondrej Stepanek, Dirk Schlüter, Dirk Reinhold, Bryen A. Jordan, Patricia Gintschel, Burkhart Schraven, Michael R. Kreutz

**Affiliations:** 1 RG Neuroplasticity, Leibniz-Institute for Neurobiology, Magdeburg, Germany; 2 Institute of Molecular and Clinical Immunology, Otto-von-Guericke University, Magdeburg, Germany; 3 Institute of Medical Microbiology, Otto-von-Guericke University, Germany; 4 Group of Adaptive Immunity, Institute of Molecular Genetics, CAS, Prague, Czech Republic; 5 Organ-Specific Immune Regulation, Helmholtz-Center of Infection Research (HZI). Braunschweig, Germany; 6 Dominick P. Purpura Department of Neuroscience, Albert Einstein College of Medicine, Bronx, New York, United States of America; 7 Department of Immune Control, Helmholtz-Center of Infection Research (HZI). Braunschweig, Germany; 8 Leibniz Group 'Dendritic Organelles and Synaptic Function', University Medical Center Hamburg-Eppendorf, Center for Molecular Neurobiology, Hamburg, Germany; Maisonneuve-Rosemont Hospital, CANADA

## Abstract

Transmembrane adaptor proteins (TRAPs) are important organisers for the transduction of immunoreceptor-mediated signals. Prr7 is a TRAP that regulates T cell receptor (TCR) signalling and potently induces cell death when overexpressed in human Jurkat T cells. Whether endogenous Prr7 has a similar functional role is currently unknown. To address this issue, we analysed the development and function of the immune system in Prr7 knockout mice. We found that loss of Prr7 partially impairs development of single positive CD4^+^ T cells in the thymus but has no effect on the development of other T cell subpopulations, B cells, NK cells, or NKT cells. Moreover, Prr7 does not affect the TCR signalling pathway as T cells derived from Prr7 knockout and wild-type animals and stimulated *in vitro* express the same levels of the activation marker CD69, and retain their ability to proliferate and activate induced cell death programs. Importantly, Prr7 knockout mice retained the capacity to mount a protective immune response when challenged with *Listeria monocytogenes* infection *in vivo*. In addition, T cell effector functions (activation, migration, and reactivation) were normal following induction of experimental autoimmune encephalomyelitis (EAE) in Prr7 knockout mice. Collectively, our work shows that loss of Prr7 does not result in a major immune system phenotype and suggests that Prr7 has a dispensable function for TCR signalling.

## Introduction

Activation of lymphocytes by antigens is the key process of adaptive immune responses. A complex interplay of proteins in the signalling pathways originating from activated immune receptors (e.g. T cell receptor, TCR; B cell receptor, BCR; Fc receptors, FcRs) is required at specific stages of lymphocyte activation. A group of proteins called transmembrane adaptor proteins (TRAPs) participate in immune receptor signalling by organizing membrane-proximal signalling complexes that are either constitutively or inducibly associated with immune receptors [1, 2]. TRAPs regulate intracellular signalling via diverse motifs (eg. SH2-, SH3- and PDZ-ligands) within their intracellular domains. To date, there are 14 members of the TRAP family (LAT, PAG/Cbp, NTAL/LAB, LIME, SIT1, TRIM, LAX, TCRζ, FcRγ, DAP10, DAP12, PRR7, SCIMP, LST1/A) [3]. LAT, the archetypal and most studied member of the TRAP family, is recruited to the TCR complex upon TCR stimulation and is indispensable for correct T cell development and optimal TCR signalling during adaptive immune responses [4, 5]. The functional importance of LAT is underscored by the severe phenotype of LAT-deficient mice, which are completely devoid of peripheral T cells [6].

Recently, we discovered and characterized a new member of the TRAP family, Proline rich 7 (Prr7) [7, 8]. Like other TRAPs, Prr7 is comprised of a short extracellular section, a single transmembrane domain and cytoplasmic region that harbours SH2, SH3, WW, and PDZ domain binding motifs. We found that TCR stimulation strongly up-regulates Prr7 expression in primary human T cells [7]. Interestingly, induced expression of Prr7 in Jurkat T cells potently stimulated production of the cytokine IL-2 and up-regulation of the T cell activation marker CD69, possibly due to increased expression of the transcription factor c-Jun. On the other hand, the TCR proximal signalling (e.g. global tyrosine phosphorylation, calcium influx) was partially inhibited. However, overexpression of Prr7 led to massive cell death within 72 hours [7]. Prr7 is highly conserved in vertebrates, which in conjunction with the striking overexpression phenotype of Prr7 in human Jurkat T cells led us to hypothesize that it might have an important functional role in TCR signalling. To test this hypothesis, we analysed the role of Prr7 in T cell developmental and function using Prr7 knockout mice.

## Results

### Prr7 is expressed in mouse immune organs and T cells

**Fig 1:**
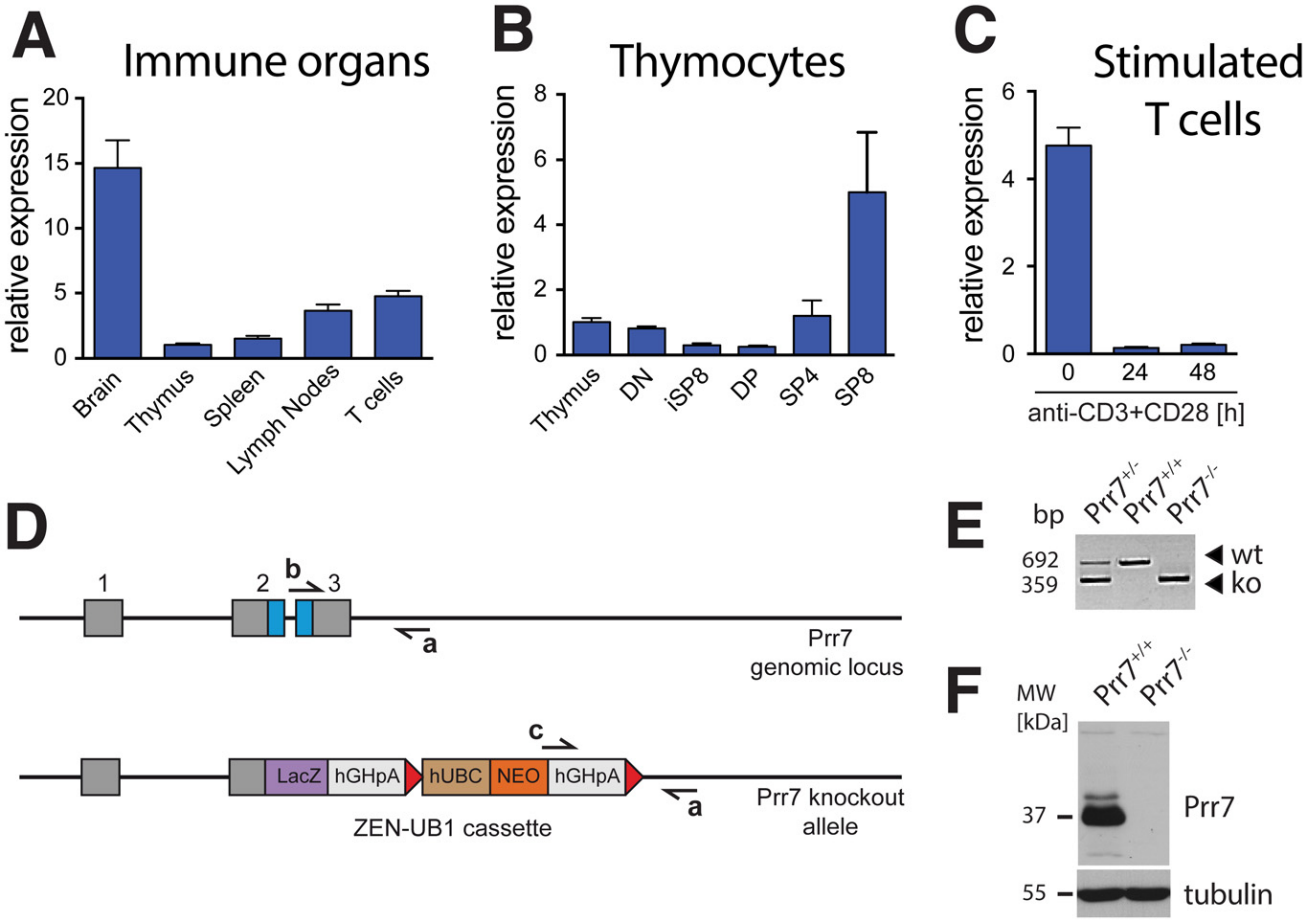
Expression analysis of Prr7 in mouse immune system and confirmation of *Prr7* gene deletion by PCR and immunoblotting. (A) qPCR analysis of Prr7 in mouse immune organs in comparison to the brain and purified T cells. The data is normalized to Gapdh and expressed relative to Prr7 levels in the thymus (expression in thymus = 1). (B) qPCR analysis of Prr7 in the thymus and purified thymocytes normalized as in (A). DN, double negative; iSP8, immature single positive cells expressing CD8; DP, double positive; SP4, CD4 single positive; SP8, CD8 single positive cells. (C) qPCR analysis of changes in Prr7 transcript levels upon stimulation of purified lymph node T cells with anti-CD3 (10 μg/ml) + anti-CD28 (1 μg/ml) for 24 h and 48 h. (D) Schematic representation of the Prr7 genomic locus, gene targeting strategy, and an approximate position of primers used for genotyping (a, b, c). LacZ, β-galactosidase, NEO, Neomycin, hUBC, human ubiquitin C promoter, hGHpA, human growth hormone polyadenylation signal sequence. Exons in the Prr7 gene are represented by grey boxes (1, 2, 3). The coding sequence spanning exons 2 and 3 is represented by blue boxes. The Neomycin gene is flanked by LoxP sites represented by red arrows. Schema not drawn to scale. (E) PCR-based mice genotyping strategy using one common reverse primer and two different forward primers specific for the Prr7 genomic locus or the ZEN-UB1 cassette as depicted in (D). (F) Immunoblotting of Prr7 protein levels in whole brain extracts from Prr7^+/+^ and Prr7^-/-^ mice. Blotting for tubulin served as a loading control. MW, molecular weight. Data in (A-C) represent the mean +SEM, n = 3.

Since the expression of Prr7 in mouse immune system has not been studied, we first analysed the mRNA levels of Prr7 in the thymus, spleen, lymph nodes, and T cells purified from lymph nodes of wild-type C57BL/6 mice. As shown in Fig 1A, relatively highest levels of Prr7 transcript were detected in lymph nodes and purified T cells. As expected, the highest Prr7 expression was observed in the cDNA from brain tissue where Prr7 is abundantly present [8]. Next, we wanted to investigate changes in the expression of Prr7 during T cell developmental stages in the thymus. To this end, we isolated double negative (DN) and double positive (DP) thymocytes, immature single positive cells expressing CD8 (iSP8), CD4 single positive (SP4), and CD8 single positive (SP8) thymocytes. Interestingly, the highest Prr7 levels were present in single positive thymocytes, particularly SP8 thymocytes (Fig 1B). In human peripheral blood T cells, Prr7 transcript increases dramatically upon TCR stimulation [7]. However, when we checked Prr7 expression in T cells purified from mouse lymph nodes and stimulated with combination of anti-CD3 and anti-CD28 for 24h and 48h we observed decrease rather than increase in Prr7 transcript levels (Fig 1C). Of note, presumably because of overall low abundance we could not detect Prr7 protein in mouse immune organs or purified T cells with currently available Prr7 antibodies by western blotting (not shown), which is in contrast to primary human T cells, where increased Prr7 protein levels can be detected upon stimulation [7].

### Mice with Prr7 gene deletion are viable and fertile

To study Prr7 function in mouse immune system, we obtained Prr7 transgenic mice generated by the KOMP consortium (www.komp.org). The targeting strategy replaces the entire Prr7 coding region by a cassette containing the LacZ gene expressed under control of the endogenous Prr7 promoter and an independently expressed Neomycin resistance gene (Fig 1D). A PCR based genotyping strategy validated the presence of the cassette in homozygous and heterozygous animals (Fig 1E). To check that Prr7 was absent at the protein level, we analysed equal amounts of total brain lysates of wild-type and knockout mice by immunoblotting with a Prr7-specific monoclonal antibody [7]. A strong band migrating at ~37 kDa was only present in samples from wild-type but not from knockout mice (Fig 1F). Prr7 deficient mice were born at normal Mendelian frequencies, without any apparent gross abnormalities and were fertile.

### Development of T and B cells are not affected by loss of Prr7

Previous work in Jurkat T cells suggested that Prr7 might be involved in pro-apoptotic processes and in regulation of c-Jun expression [7]. Apoptosis is a fundamental process of T cell biology [9]. During their multistep development in the thymus, the vast majority of developing T cells are removed through negative and positive selection [10]. Interestingly, c-Jun is required in T cell development for successful β-selection in the thymus, a process by which T cells acquire β chains for their TCRs. In T cell specific c-Jun knockout mice, T cell development is partially arrested at the third double-negative stage (DN3) [11].

**Fig 2:**
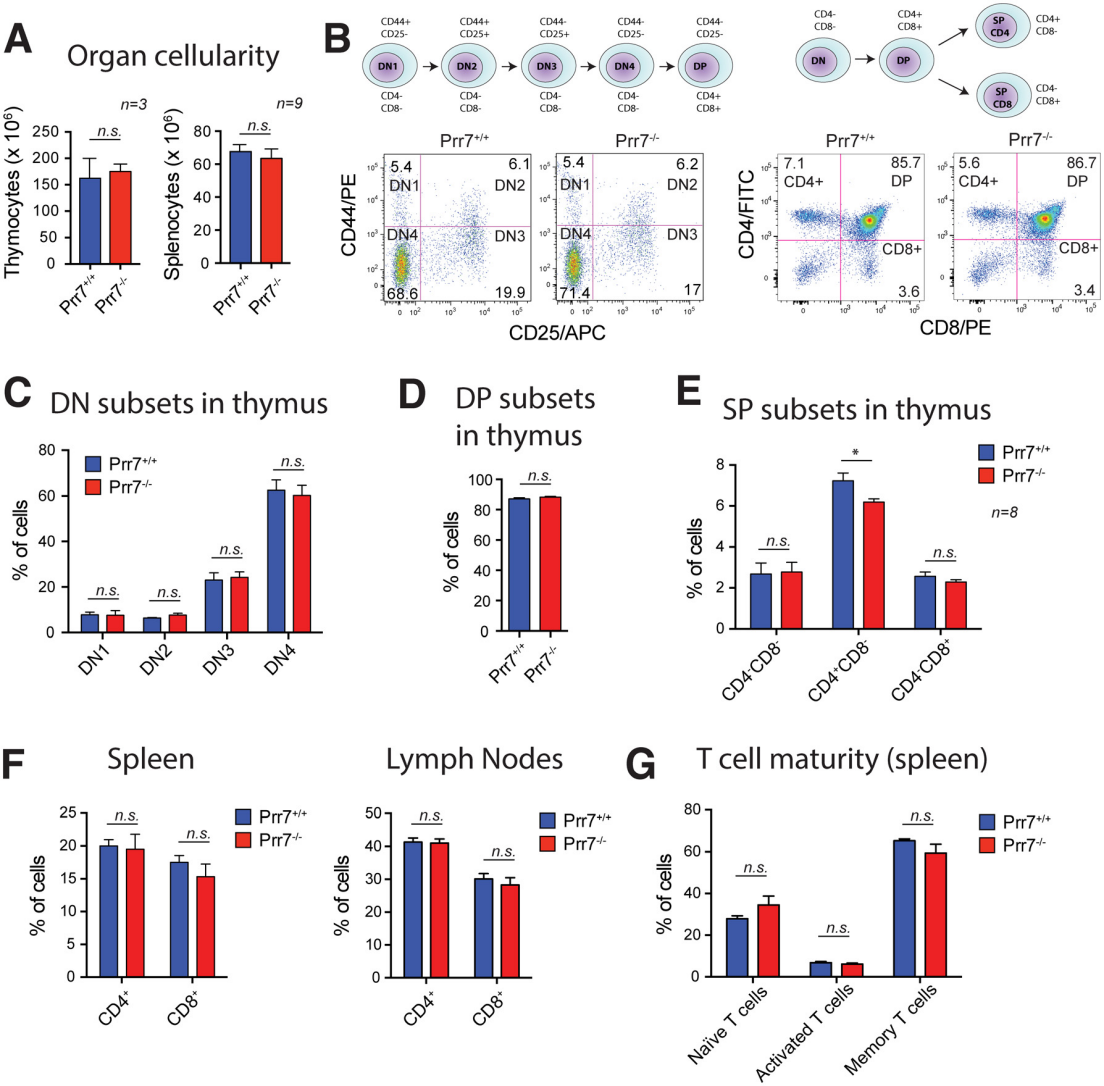
T cell development is largely unaffected in Prr7-deficient mice. (A) Total numbers of nucleated cells in the thymus (left) and spleen (right) isolated from Prr7^+/+^ and Prr7^-/-^ mice as counted using a haemocytometer. (B) Schematic representation of T cell developmental stages in the thymus. DN, double negative, DP, double positive, SP, single positive. Lower panels with dot plots are representative examples of flow cytometry analysis of thymocyte subpopulations. Percentages of DN subpopulations (C), DP subpopulations (D), and SP subpopulations (E) in thymi of Prr7^+/+^ and Prr7^-/-^ mice as analysed by flow cytometry. (F) Flow cytometry analysis of CD4^+^ and CD8^+^ T cells subpopulations in the secondary lymphatic organs spleen and lymph nodes expressed as percentage of total. (G) Flow cytometry analysis of splenic CD3^+^ T cells expressing markers of naïve T cells (CD62^+^CD25^-^), activated T cells (CD62L^-^CD25^+^), or memory T cells (CD62L^-^CD25^+^). Data in (A-G) represent the mean +SEM, n = 3–8. *p < 0.05, n.s., not significant.

We therefore assessed whether Prr7^-/-^ T cells would successfully pass through all developmental stages in the thymus and populate secondary lymphatic organs. To address this question, we first examined the total number of nucleated cells in the thymus and spleen. The analysis revealed that the cellularity of these organs in both wild-type and Prr7-deficient mice was comparable (Fig 2A). Accordingly, further analysis of T cell development in the thymus using flow cytometry and four T cell surface markers (CD4, CD8, CD44, and CD25), used to distinguish the major developmental stages of double negative (DN) and single positive (SP) T cells, confirmed that T cells develop normally in the absence of Prr7 (Fig 2B). Except for a minor, but statistically significant decrease in CD4 single positive population in the thymi of Prr7^-/-^ mice, no apparent differences in T cell development between wild-type and knockout were detected (Fig 2C, 2D and 2E).

Mature single positive (CD4^+^ or CD8^+^) T cells migrate to and populate secondary lymphatic organs. To analyse whether Prr7 deficiency might interfere with this process we measured the composition of T cell subpopulations in the spleen and lymph nodes. We found the same percentage of CD4^+^ or CD8^+^ cells in spleen and lymph nodes isolated from Prr7^+/+^ and Prr7^-/-^ mice (Fig 2F). In secondary lymphatic organs, the maturity of CD4^+^ T cells can be categorised by the presence of the surface markers CD62L and CD25. Again, the percentage of naïve T cells (CD62L^+^CD25^-^), activated T cells (CD62L^-^CD25^+^), and memory T cells (CD62^-^CD25^-^) was unaffected by the absence of Prr7 (Fig 2G). Moreover, numbers of γ/δ T cells (CD3^+^TCRγδ^+^), subpopulation of T cells enriched in regulatory T cells (CD4^+^CD25^+^), natural killer (NK) cells (NK1.1^+^TCRβ^-^), and NK T cells (NK1.1^+^TCRβ^+^) were identical in the spleen and lymph nodes of Prr7^+/+^ and Prr7^-/-^ mice (S1A and S1B Fig). Finally, B cell development and maturation throughout the bone marrow and spleen (S1C Fig) and myeloid and lymphoid dendritic cell (DC) numbers in the spleen (data not shown) were normal in Prr7 knockout mice. Collectively these data do not support a major role for Prr7 in T cell and B cell development.

### Prr7 is dispensable for TCR signalling

**Fig 3:**
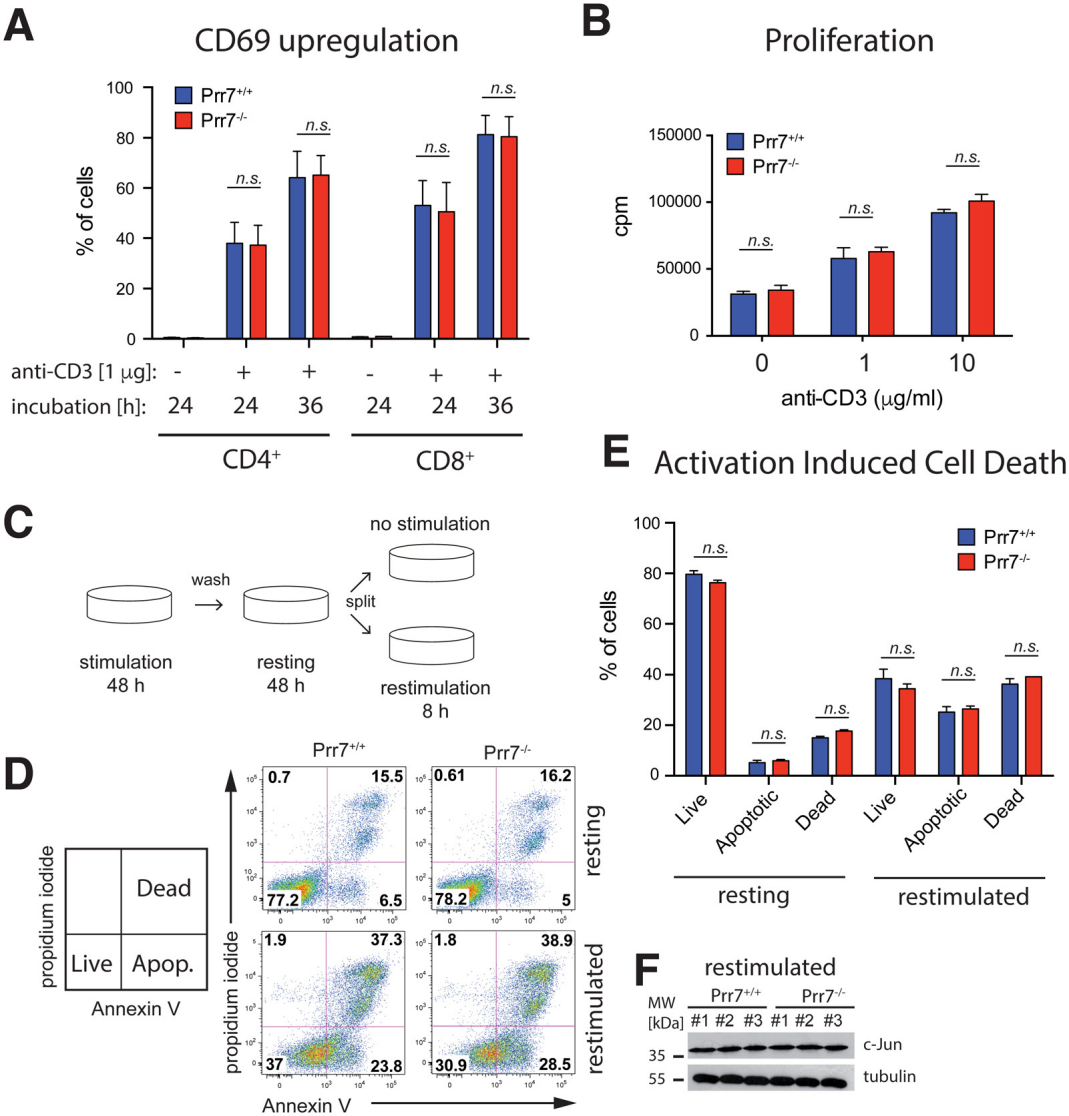
TCR response and AICD is unaffected in T cells from Prr7-deficient mice. (A) Flow cytometry analysis of the activation marker CD69 in Prr7-deficient CD4^+^ or CD8^+^ T cells stimulated with 1 μg of plate-bound anti-CD3 for 24 or 36 h. (B) Proliferation of Prr7^+/+^ and Prr7^-/-^ splenocytes in response to TCR stimulation with plate-bound anti-CD3 measured as [^3^H]thymidine incorporation (DNA synthesis). cpm, counts per minute. (C) Schema of the *in vitro* AICD induction protocol. (D) Representative examples of flow cytometry analysis of AICD in T cells upon restimulation. Live = PI^-^Annexin V^-^, Apoptotic = PI^-^Annexin V^+^, Dead = PI^+^Annexin V^+^ (E) Quantification and statistical analysis of AICD performed as shown in (D). (F) Immunoblotting of c-Jun total levels in restimulated T cells isolated from three different wild-type and knockout mice (#1, #2, #3). Tubulin served as a loading control. Data in (A, B, D) represent the mean + SEM of at least three animals per group. n.s., not significant.

Since Prr7 overexpression in Jurkat T cells potently affected TCR signalling [7], we next tried to identify a potential role for Prr7 in TCR signalling in primary mouse T cells. To this end, we first isolated total splenocytes from Prr7^+/+^ and Prr7^-/-^ mice, stimulated them with anti-CD3 to trigger the TCR and measured up-regulation of the T cell activation marker CD69 in both CD4^+^ and CD8^+^ T cells. As depicted in Fig 3A and S2 Fig, T cells from wild-type and Prr7 knockout mice expressed the same level of CD69 24 h and 36 h following stimulation, indicating that TCR signalling is largely unaffected by the absence of Prr7.

As an additional test of the TCR response, we then probed the capacity of T cells to proliferate in response to TCR stimulation. Here, we stimulated splenocytes with increasing amounts of immobilized anti-CD3 for 72 h, pulse labelled them for the last 16 h with [^3^H]thymidine, and measured radioisotope incorporation as an index of lymphocyte proliferation. This test revealed that proliferation of T cells derived from wild-type and Prr7-deficient mice was similar at all anti-CD3 concentrations tested (Fig 3B).

### Prr7 does not regulate activation-induced cell death of mouse T cells

Our previous work showed that Prr7 overexpression induces massive cell death in Jurkat T cells [7]. Since Prr7 expression levels change in activated T cells, we hypothesized that endogenous Prr7 might regulate activation induced cell death (AICD), a negative regulatory process by which activated T cells are removed at the end of an adaptive immune response [12, 13]. Given that Prr7 overexpression in T cells promotes cell death, we speculated that its absence might diminish or delay the onset of the AICD apoptotic programme. To address this possibility, we established an *in vitro* AICD model. Briefly, T cells isolated from wild-type and Prr7-deficient mice were stimulated for 48 h with anti-CD3 and anti-CD28, washed and left to rest for an additional 48 h in the presence of exogenous IL-2. To trigger apoptosis, the cells were exposed again to the same concentrations of anti-CD3 and anti-CD28 for 8 h in the absence of exogenous IL-2. Apoptosis was measured by combination of fluorescently labelled Annexin V, which specifically binds apoptotic cells and the cell impermeable DNA dye propidium iodide (PI). Surprisingly, T cells from both wild-type and Prr7 knockout mice responded in the same way and the number of apoptotic and dead cells was not statistically significantly different in the restimulated cultures (Fig 3C and 3E). Thus, the lack of Prr7 has no effect on cell death in this AICD model.

Upon expression of Prr7 in Jurkat T cells, the transcription factor c-Jun was strongly upregulated, which coincided with the onset of cell death in Prr7 expressing cells [7]. Therefore, we checked c-Jun expression in restimulated T cell in AICD experiments by Western blotting but found no difference in total c-Jun levels between Prr7-deficient and wild-type mice (Fig 3F).

### Normal pathogen control and T cell response in *Listeria monocytogenes*-infected Prr7-deficient mice

**Fig 4:**
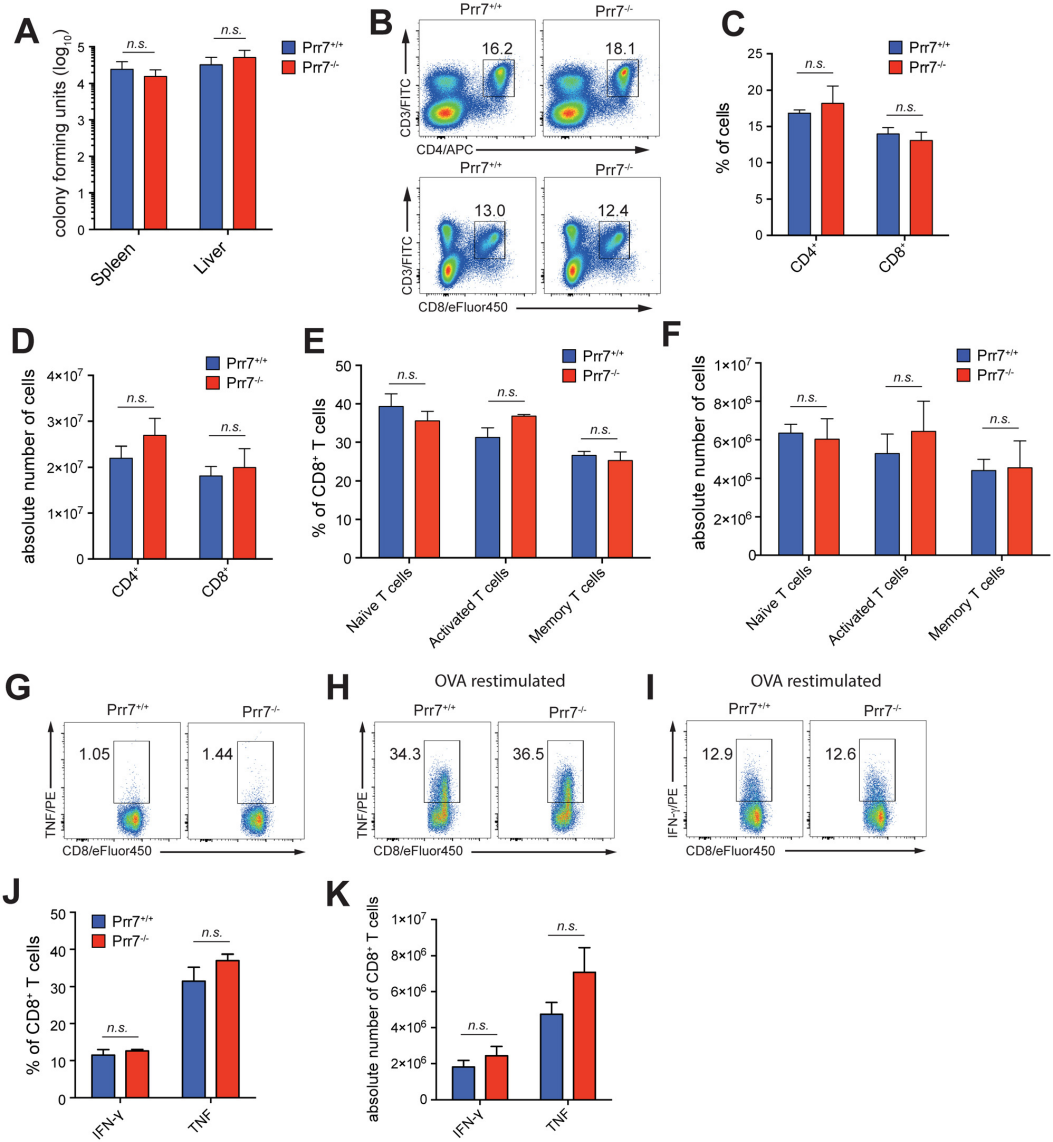
Prr7 deficiency does not influence T cell response to *Listeria monocytogenes* infection. (A) *Prr7*^-/-^ mice and *Prr7*^+/+^ control mice were i.v. infected with 1x10^4^ Lm ova. On day 9 post infection, colony forming units were determined in spleen and liver. (B) Representative dot plot of CD4^+^ and CD8^+^ T cells in the spleen of infected mice. (C) Frequency of CD4^+^ and CD8^+^ T cells in the spleen of infected mice. (D) Absolute number of CD4^+^ and CD8^+^ T cells in the spleen of infected mice. (E) Frequency of CD8^+^ naive (CD62L^+^CD44^-^), activated (CD62L^-^CD44^+^) and memory (CD62L^+^CD44^+^) T cells in the spleen of infected mice. (F) Absolute number of CD8^+^ naive, activated and memory T cells in the spleen of infected mice. (G-K) Splenocytes of infected mice were restimulated with Ova_257-264_-peptide (SIINFEKL, 10^−8^ M) or left unstimulated for 12 h in the presence of Brefeldin A, to allow for the intracellular accumulation of cytokines. (G) Dot plot of IFN-γ and TNF-producing CD8^+^ T cells without restimulation. (H) Dot plot of TNF-producing CD8^+^ T cells after restimulation. (I) Dot plot of IFN-γ producing CD8^+^ T cells after restimulation. (J) Frequency of IFN-γ and TNF-producing CD8^+^ T cells in the spleen of infected mice. (K) Absolute number of IFN-γ and TNF-producing CD8^+^ T cells in the spleen of infected mice. Data are represented as mean ± SEM of 4–5 mice per group. n.s. not significant.

*Listeria monocytogenes* is a facultative intracellular Gram-positive bacterium invading the spleen and liver. The containment and clearance of *Listeria monocytogenes* requires both innate and adaptive immune responses with a pivotal role for NK cells, macrophages, dendritic cells, and CD8^+^ T cells [14]. For this reason, this informative non-lethal infection model is often used to screen for the functionality of cellular immune responses *in vivo* [15]. We intravenously challenged Prr7^-/-^ and control Prr7^+/+^ mice with ovalbumin (OVA)-expressing *Listeria monocytogenes* (Lm ova). After 9 days, i.e. at the peak of the primary *Listeria*-specific CD8^+^ T cell response [16], we measured the number of bacteria present in the spleen and liver in dissociated organs as number of colony forming units (CFU). As shown in Fig 4A, there was no difference between spleens and livers of wild-type and Prr7-deficient mice. In addition, we found that similar numbers of CD4^+^ and CD8^+^ T cells were recruited to spleens (Fig 4B–4D) or livers (S3A–S3C Fig) of Prr7^-/-^ and control Prr7^+/+^ mice. Since CD8^+^ T cells play a critical role in eradicating intracellular pathogens and are also the most effective T cell subset mediating the protection against *Listeria* [17], we further analysed the composition of CD8^+^ subpopulations in spleens and livers of *Listeria*-infected mice. Subsequent experiments utilizing a combination of T cell surface markers CD62L/CD44 and flow cytometry revealed that the cell number of naïve (CD62L^+^CD44^-^), activated (CD62L^-^CD44^+^) and memory (CD62L^+^CD44^+^) CD8^+^ T cells did not differ in the spleen (Fig 4E and 4F) and liver (S3D and S3E Fig) of infected mice of both genotypes. In addition, loss of Prr7 did not influence the relative and absolute numbers of OVA_257-264_-specific TNF and IFN-γ-producing CD8+ T cells in spleen (Fig 4G–4K) and liver (S3F–S3I Fig).

The results of the *Listeriosis* infection model suggest that Prr7 deficiency does not impair the ability of the mouse immune system to cope with *Listeria monocytogenes* infection. Moreover, Prr7 appears to be fully dispensable for the recruitment of T cells to the infected spleen and liver and their activation by the bacteria *in vivo*.

### Normal effector functions of Prr7-deficient T cells in mouse Experimental Autoimmune Encephalomyelitis (EAE)

**Fig 5:**
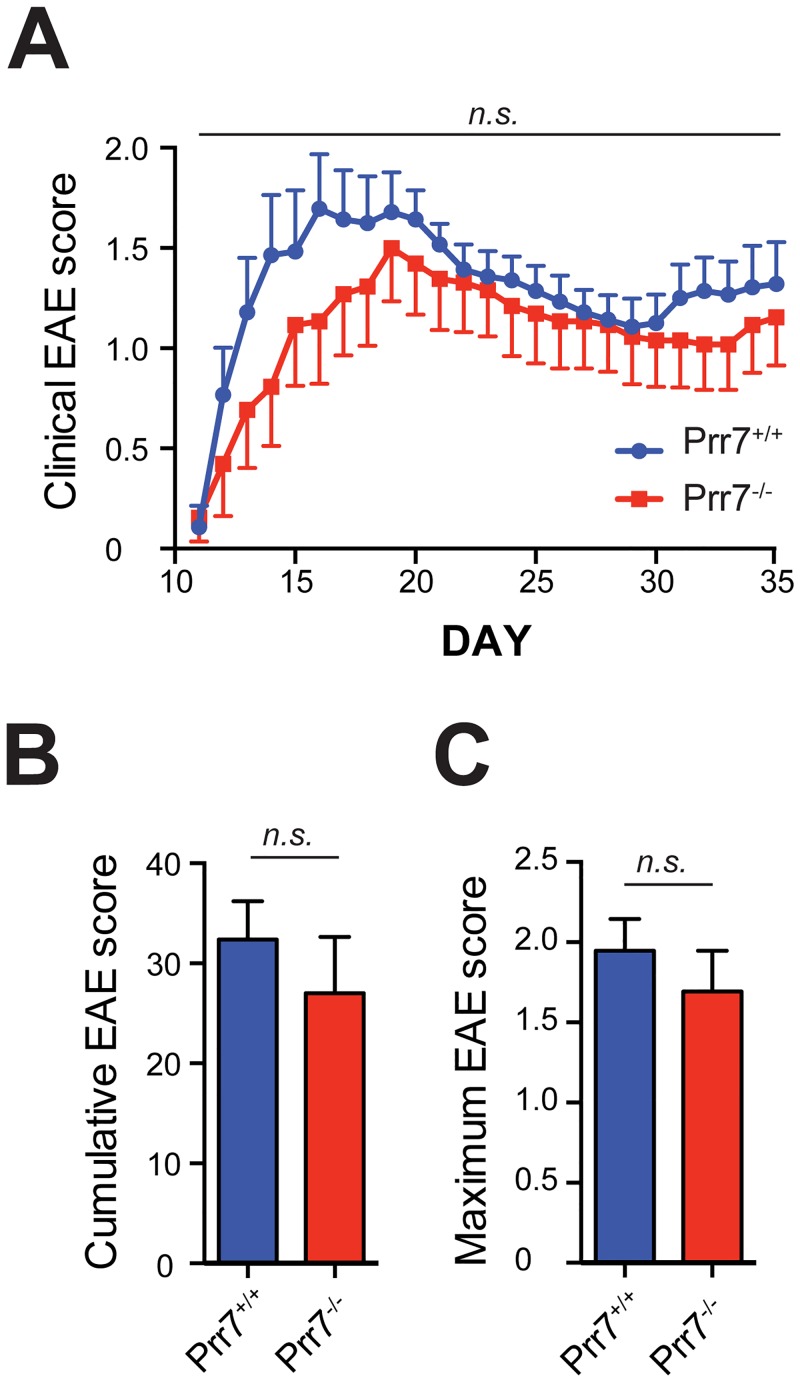
Absence of Prr7 does not affect the susceptibility of mice to EAE. (A) Clinical EAE scores in 8–12 week old WT and Prr7 knockout female mice upon immunization with MOG peptide over time. (B) Cumulative EAE score from (A). (C) Maximum EAE scores from (A). Data are represented as mean +/- SEM of 13 (Prr7^-/-^) and 14 (Prr7^+/+^) mice per group from two independent experiments. n.s. not significant.

Experimental autoimmune encephalomyelitis (EAE) is a widely used animal model of the autoimmune disease Multiple Sclerosis (MS) [18–20]. In MS, an inflammatory immune reaction to central nervous system antigens in the periphery leads to chronic myelin damage and neurodegeneration [21, 22]. As T cells (T_H_1 and T_H_17 CD4^+^ T cell subsets in particular) play a central role in the pathogenesis of EAE [23, 24], we used this model to further look into a possible role of Prr7 in CD4^+^ T cell effector functions (activation, migration, and reactivation) *in vivo*. To this end, we immunised Prr7 knockout and matched wild-type control female mice with myelin oligodendrocyte glycoprotein (MOG) peptides (Day 0 and 2) and monitored the spontaneous development of clinical signs of EAE for 35 days (Fig 5A–5C). Although Prr7^-/-^ mice tend to develop a slightly milder EAE than wild-type mice, the differences were not statistically significant.

## Discussion

In this report, we provide a concise analysis of immune system function in Prr7-deficient mice. The central aim of the study was to reveal a potential function of Prr7 in TCR signalling *in vitro* and *in vivo*.

Previously, we identified Prr7 as a TRAP expressed in activated T cells [7]. Interestingly, the analysis of Prr7 expression in mouse lymph node T cells revealed an opposite effect of the TCR stimulation on Prr7 transcript levels, i.e. downregulation instead of upregulation of Prr7 mRNA observed in human T cells. The reason for such a difference is unknown. In a follow-up study, it would be informative to compare Prr7 expression in activated T cells from different mouse organs and blood as well as effect of different TCR stimuli. In addition, the Prr7 expression pattern in mouse immune tissues and in thymocytes seems to positively correlate with the number of mature T cells in the organs and T cell maturity within the thymus with relatively highest Prr7 expression in lymph nodes and in CD8 single positive thymocytes.

In our previous study, PRR7 potently regulated TCR signalling and cell death when overexpressed in Jurkat T cell line [7]. These *in vitro* data prompted us to focus our analysis on T cells in knockout mice. Unexpectedly, we found that T cell development and effector functions remain largely unaffected in the absence of Prr7. Prr7 deficiency resulted only in a partial impairment of thymic development of single positive CD4^+^ T cells. However, such a minor decrease in CD4^+^ T cells was not reflected in periphery (spleen, lymph nodes) and the functional relevance *in vivo* is questionable since Prr7-deficient mice mounted an effective immune response to *Listeria monocytogenes* infection and readily developed autoimmunity in the EAE model.

The lack of an overt immune phenotype in Prr7 knockout mice resembles the outcome of studies with knockout mice of several other TRAPs. For instance, mice deficient in PAG1, a CSK scaffold and TRAP abundantly expressed in all immune cell types with strong *in vitro* evidence for a negative regulatory role in immunoreceptor signalling, show basically no immune phenotype [25, 26]. Nonetheless, varying protein levels of PAG1 in different human malignant diseases correlate with oncogenic Src activity, suggesting its physiological function might be revealed only under special circumstances like cell transformation [27]. The same might be true for Prr7, since it also interacts with Src [7]. Moreover, it should be noted that the abundance of Prr7 is very low in T cells as compared to brain where the protein couples N-Methyl-D-Aspartate-signalling to c-Jun dependent gene transcription and neuronal cell death [28]. The lack of an overt immune phenotype is therefore not completely surprising.

Similarly, only minimal or mild phenotypes were found in mice deficient in LIME1 [29], TRAT1 (TRIM) [30], SIT1 [31], GAPT [32], and LAX1 [33]. Interestingly, combined deficiency of TRAT1 and SIT1 leads to a more severe phenotype in T cell development in the thymus [34]. Thus, the relatively mild phenotypes found in TRAP knockout mice could be explained by the existence of compensatory mechanisms and functional redundancy. Consistent with this notion, interactions between TRAPs may regulate certain specific immune processes. For example, mast cells deficient in the TRAP LAT2 (NTAL/LAB) are hyper-reactive to FcεRI stimulation, while mast cells deficient in LAT display an opposite phenotype. However, the combined deficiency of LAT and LAT2 leads to more severe inhibition of FcεRI signalling than LAT deficiency alone [35].

It is tempting to speculate that another TRAP is able to compensate for the lack of Prr7 in knockout mice or that several proteins with overlapping function contribute to the same biological process. Moreover, although the strong overexpression phenotype of Prr7 suggested that Prr7 is a TRAP in T cells regulating TCR signalling and cell death [7], these results may represent an example of how *in vitro* experiments do not predict function *in vivo*.

## Conclusions

In summary, the analysis of Prr7 knockout mice did not reveal major defects in the immune system but this does not preclude the possibility that an even more detailed analysis might identify a function in cellular processes of immune cells that were not investigated in this study.

## Materials and Methods

### Mice

The Prr7 knockout mice were obtained from KOMP (www.komp.org) and housed in the animal facilities of the Leibnitz Institute for Neurobiology, Magdeburg or at the Medical Faculty of the Otto-von-Guericke-University, Magdeburg. The Prr7 knockout mice were generated on the C57BL/6 background. All procedures were carried out in strict accordance with the protocols approved by the Institutional Animal Care and Use Committee of the LIN and the Landesverwaltungsamt Dessau.

### Cell isolation, culture, and stimulation

Mouse thymic, splenic and lymph node cells for *in vitro* studies were obtained by passing the isolated organs through 70 μm cell strainers. The red blood cells contaminating spleen samples were lysed with ammonium chloride buffer. T cells were purified by negative selection using magnetic cell sorting (Total T cell isolation kit, Miltenyi Biotec). Thymic subpopulations were isolated by FACS sorting as follows: all CD45^+^ and then DN (CD4^-^CD8^-^), iSP8 (CD4^-^CD8^+^TCR^int^), DP (CD4^+^CD8^+^), SP4 (CD4^+^CD8^-^), SP8 (CD4^-^CD8^+^TCR^hi^). Where needed, isolated cells were cultured in RPMI 1640 medium supplemented with foetal bovine serum (10% v/v) and penicillin/streptomycin. Hepatic leukocytes were separated by Percoll gradient centrifugation (GE Healthcare, Freiburg, Germany). For T cell stimulation, the isolated cells were plated on culture dishes pre-coated overnight with anti-CD3 (diluted 1–10 μg/ml in PBS) and soluble anti-CD28 was added to the culture media at concentration 1 μg/ml.

### Antibodies and reagents

The following antibodies and reagents were used for flow cytometry according to the manufacturers’ instructions: anti-CD3ε-FITC (553061, BD or 145-2C11, eBioscience), anti-CD4-FITC (553729, BD), anti-CD4-Alexa488 (100529, BioLegend), anti-CD4-APC (RM4-5, eBioscience), anti-CD5-biotin (553019, BD), anti-CD8-PE (553033, BD), anti-CD8-PacBlue (100725, BioLegend), anti-CD8-eFluor450 (53–6.7, eBioscience), anti-CD11c-APC (117310, BioLegend), anti-CD16/32 (Fc block, 101320, BioLegend), anti-CD21-FITC (123407, BioLegend), anti-CD23-biotin (101603, BioLegend), anti-CD25-Alexa647 (102020, BioLegend), anti-CD44-PE (103008, Biolegend), anti-CD45-PerCP (30-F11, BioLegend), CD62L-PE (553151, BD), anti-CD69-APC (104513, BioLegend), anti-B220-FITC (103205, Biolegend), anti-IgD-FITC (553439, BD), anti-IgM-PE (553409, BD), anti-IFN-γ (XMG1.2, BD Biosciences), anti-NK1.1-FITC (108705, BioLegend), anti-TCRβ-PE (109208, BioLegend), anti-TCRγδ-APC (118116, Biolegend), anti-TNF-PE (MP6-XT22, BD Biosciences), Streptavidin-Dy649 (405224, BioLegend), Annexin-V/FITC (640905, BioLegend), (7-AAD (420403, BioLegend), propidium iodide (Sigma Aldrich). For cell stimulations the following antibodies and reagents were used: anti-CD3 (clone 145–2C11), anti-CD28 (102111, BioLegend), mouse IL-2 (575402, BioLegend). Antibodies used for western blotting were obtained from the following sources: anti-Prr7 (TRAP3/10, Exbio), c-Jun (60A8, 9165P, Cell Signaling), and anti-α-tubulin III (Sigma Aldrich). Additional experiment specific antibodies are also listed in other sections.

### Flow cytometry

After blocking the Fc receptors with 0.5 μg anti-CD16/32 1x10^6^ cells per sample were stained with conjugated primary antibodies for 30 min on ice in cell staining buffer composed of PBS, 0.5% BSA, and 0.1% NaN_3_. The samples were analysed using LSRFortessa Cell Analyser or FACS Canto II (BD Biosciences) and FlowJo data analysis software (FlowJo). Dead cells stained with live cell impermeable DNA dyes 7-AAD or propidium iodide (PI) were excluded from analysis. Intracellular stainings were performed after *ex vivo* restimulation for 12 h at 37°C and 5% CO_2_ in the presence of Brefeldin A. Cells were fixed and permeabilized with Cytofix/Cytoperm Kit according to manufacturer’s manual (BD Biosciences).

### Genotyping PCR

Crude DNA isolated from mouse tail biopsies was amplified with a common reverse primer (5’-AAG CCC TTG AGA AAC AAC CTT-3’) and two forward primers specific for the Prr7 genomic locus (5’-ACA TGT CTA AGC CGC CGT GCT A-3’) or the ZEN-UB1 cassette (5’-GTT TTG CCA AGT TCT AAT TCC A-3’) using Taq DNA polymerase (201203, Qiagen) according to manufacturer’s protocol.

### qPCR

Total RNA from dissected organs, purified cells or brain (frontal cortex) was extracted using Mini RNA Kit (Zymo Research) with on-column DNAse treatment. Subsequently, 1 μg of isolated RNA was reverse transcribed (ReverseAid, Thermo Scientific) with anchored oligo(dT)_20_ and random pentadecamers. qPCR was performed using SYBR Green chemistry (Roche) and LC480 Light Cycler (Roche) with extensively validated Prr7 and Gapdh specific primers (Prr7 F: GAC GAG TTC GAA GAG GAT GC, Prr7 R: GAG GGG CAA CTG TGG TTC, Gapdh F: ATG GTG AAG GTC GGT GTG A, Gapdh R: AAT CTC CAC TTT GCC ACT GC) in technical replicates. The relative Prr7 expression levels were calculated in Excel (Microsoft) using ΔΔCt method.

### Brain protein extraction

The whole brain was homogenized and the crude membrane fraction (P2 fraction) isolated by centrifugation and lysed in RIPA buffer as described before [8].

### Immunoblotting

Protein samples were resolved by SDS-PAGE and transferred to PVDF membranes. The proteins were detected by primary antibodies and visualized by chemoluminiscence using secondary HRP-conjugated antibodies.

### T cell proliferation assay (DNA synthesis)

Proliferation of mouse splenocytes was assessed by measuring [^3^H]thymidine [^3^H-TdR] incorporation. [^3^H]thymidine was added at 0.2 μCi/well for the last 16 h of the incubation. At the end of the incubation period, all cells were harvested and radioisotope incorporation was measured as an index of lymphocyte proliferation using a Betaplate liquid scintillation counter (MicroBeta, Wallac, Turku, Finland).

### Listeriosis infection model

**Infection.** Prr7^-/-^ and Prr7^+/+^ mice (8- to 12-week old) were intravenously infected with 1x10^4^ ovalbumin-expressing *Listeria monocytogenes* (Lm ova). Animals were sacrificed on day 9 post infection. **Colony forming units (CFU).** Single cell suspensions from the spleen and liver were prepared by passing the organs through a 70 μM cell strainer. Ten-fold serial dilutions were plated on heart-brain agar and incubated for 24 h at 37°C. Bacterial colonies were counted microscopically.

### Experimental autoimmune encephalomyelitis (EAE)

**Peptides.** Myelin oligodendrocyte glycoprotein peptide 35–55 (MOG p35-55), corresponding to mouse sequence (MEVGWYRSPFSRVVHLYRNGK), was synthesized on a peptide synthesizer by standard 9-fluorenylmethoxycarbonyl chemistry, and purified by high-performance liquid chromatography (HPLC). **Induction, treatment and evaluation of active EAE.** Female Prr7^-/-^ and control Prr7^+/+^ mice (8- to 12-week old) were immunized subcutaneously (s.c.) in depots distributed over 4 spots across the flanks with 200 μg MOG (p)35-55 in 0.2 ml emulsion consisting of equal volumes of PBS and complete Freund's adjuvant (CFA; Sigma, Germany), containing 4 mg/ml of mycobacterium tuberculosis H37Ra (Difco, Detroit, MI). 200 ng pertussis toxin (PTX; List Biological Laboratories, Campbell, CA) was administered intraperitoneally (i.p.) at days 0 and 2. The mice were scored daily for clinical signs of EAE according to the following increasing severity scale: 0: no disease; 1: tail weakness (tail plegia); 2: hindlimb paraparesis and/or weak rightning-reflex; 3: hindlimb paraplegia; 4: paraplegia with forelimb weakness or paralysis; 5: moribund animals. Mice with intermediate clinical signs were scored in 0.5 increments. Daily clinical scores were calculated as the average of all individual disease scores of each group.

### Statistical analysis

For experiments with statistical analysis, a two-tailed Student’s t test was performed, and p values less than or equal to 0.05 were considered significant. Statistical comparison of EAE disease severity was accomplished by a non-parametric Mann-Whitney test as described previously [36]. The data were analysed using Prism 6 statistical software (GraphPad).

## Supporting Information

S1 Fig Unaffected B cell development in Prr7-deficient mice. (A) Flow cytometry analysis of γ/δ T cell and enriched in regulatory T cells (CD4^+^CD25^+^) subpopulations in the lymph nodes. (B) Flow cytometry analysis of NK (NK1.1^+^TCRβ^-^) and NK T (NK1.1^+^TCRβ^+^) cells in the spleen stained with anti-NK1.1 and anti-TCRβ. (C) No gross defects were found in B cell development in Prr7 deficient animal. The upper schema depicts the main developmental stages of B cells in the bone marrow and in the spleen. MZ, marginal zone B cells. The middle and lower panels are the results of flow cytometry analysis of B cell developmental stages in the bone marrow and in the spleen of *Prr7*^-/-^ mice and control *Prr7*^+/+^ mice defined as follow: Immature B cells (B220^lo^IgM^+^), Mature B cells (B220^hi^IgM^+^), Pro & Pre B cells (B220^+^IgM^-^), T1 B cells (CD23^+^CD21^+^IgM^+^), T2 B cells (CD23^+^CD21^+^IgM^+^), MZ B cells (CD23^-^CD21^+^B220^+^), Immature B cells (IgM^hi^IgD^lo^), Mature B cells (IgM^lo^IgD^hi^). Data in (A, B, C) represent the mean + SEM of at least three animals per group. n.s., not significant.
(TIF)

S2 Fig (A) Data from Fig 3A expressed as the mean fluorescent intensities (MFI) of CD69 antibody staining. Data in represent the mean + SEM of minimum three animals per group. n.s., not significant.
(TIF)

S3 Fig Prr7 deficiency does not influence T cell response to *Listeria monocytogenes* infection. (A) Representative dot plot of CD4^+^ and CD8^+^ T cells in liver of Lm ova-infected mice. (B) Frequency of CD4^+^ and CD8^+^ T cells in liver of infected mice. (C) Absolute number of CD4^+^ and CD8^+^ T cells in liver of infected mice. (D) Frequency of CD8^+^ naive (CD62L^+^CD44^-^), activated (CD62L^-^CD44^+^) and memory (CD62L^+^CD44^+^) T cells in liver of infected mice. (E) Absolute number of CD8^+^ naive, activated and memory T cells in liver of infected mice. (F-I) Hepatic leukocytes of infected mice were restimulated with Ova_257-264_-peptide (SIINFEKL, 10^−8^ M) for 12 h in the presence of Brefeldin A. (F) Dot plot of TNF-producing CD8^+^ T cells. (G) Dot plot of IFN-γ producing CD8^+^ T cells. (H) Frequency of IFN-γ and TNF-producing CD8^+^ T cells in spleen of infected mice. (I) Absolute number of IFN-γ and TNF-producing CD8^+^ T cells in spleen of infected mice. Data are represented as mean + SEM of 3–4 mice per group. n.s. not significant.
(TIF)

## References

[1] Horejsi V, Zhang W, Schraven B. Transmembrane adaptor proteins: organizers of immunoreceptor signalling. Nat Rev Immunol. 2004;4(8): 603–616. 10.1038/nri1414 15286727

[2] Simeoni L, Lindquist JA, Smida M, Witte V, Arndt B, Schraven B. Control of lymphocyte development and activation by negative regulatory transmembrane adapter proteins. Immunol Rev. 2008;224: 215–228. 10.1111/j.1600-065X.2008.00656.x 18759929

[3] Stepanek O, Draber P, Horejsi V. Palmitoylated transmembrane adaptor proteins in leukocyte signaling. Cell Signal. 2014;26(5): 895–902. 10.1016/j.cellsig.2014.01.007 24440308

[4] Fuller DM, Zhang W. Regulation of lymphocyte development and activation by the LAT family of adapter proteins. Immunol Rev. 2009;232(1): 72–83. 10.1111/j.1600-065X.2009.00828.x 19909357 PMC3646374

[5] Balagopalan L, Coussens NP, Sherman E, Samelson LE, Sommers CL. The LAT story: a tale of cooperativity, coordination, and choreography. Cold Spring Harb Perspect Biol. 2010;2(8): a005512 10.1101/cshperspect.a005512 20610546 PMC2908767

[6] Zhang W, Sommers CL, Burshtyn DN, Stebbins CC, DeJarnette JB, Trible RP, et al Essential role of LAT in T cell development. Immunity. 1999;10(3): 323–332. 10204488 10.1016/s1074-7613(00)80032-1

[7] Hrdinka M, Draber P, Stepanek O, Ormsby T, Otahal P, Angelisova P, et al PRR7 is a transmembrane adaptor protein expressed in activated T cells involved in regulation of T cell receptor signaling and apoptosis. J Biol Chem. 2011;286(22): 19617–19629. 10.1074/jbc.M110.175117 21460222 PMC3103341

[8] Murata Y, Doi T, Taniguchi H, Fujiyoshi Y. Proteomic analysis revealed a novel synaptic proline-rich membrane protein (PRR7) associated with PSD-95 and NMDA receptor. Biochem Biophys Res Commun. 2005;327(1): 183–191. 10.1016/j.bbrc.2004.11.154 15629447

[9] Zhang N, Hartig H, Dzhagalov I, Draper D, He YW. The role of apoptosis in the development and function of T lymphocytes. Cell Res. 2005;15(10): 749–769. 10.1038/sj.cr.7290345 16246265

[10] Carpenter AC, Bosselut R. Decision checkpoints in the thymus. Nat Immunol. 2010;11(8): 666–673. 10.1038/ni.1887 20644572 PMC3388799

[11] Riera-Sans L, Behrens A. Regulation of alphabeta/gammadelta T cell development by the activator protein 1 transcription factor c-Jun. J Immunol. 2007;178(9): 5690–5700. 17442952 10.4049/jimmunol.178.9.5690

[12] Krammer PH, Arnold R, Lavrik IN. Life and death in peripheral T cells. Nat Rev Immunol. 2007;7(7): 532–542. 10.1038/nri2115 17589543

[13] Brenner D, Krammer PH, Arnold R. Concepts of activated T cell death. Crit Rev Oncol Hematol. 2008;66(1): 52–64. 10.1016/j.critrevonc.2008.01.002 18289867

[14] Pamer EG. Immune responses to Listeria monocytogenes. Nat Rev Immunol. 2004;4(10): 812–823. 10.1038/nri1461 15459672

[15] Lara-Tejero M, Pamer EG. T cell responses to Listeria monocytogenes. Curr Opin Microbiol. 2004;7(1): 45–50. 10.1016/j.mib.2003.12.002 15036139

[16] Busch DH, Pilip IM, Vijh S, Pamer EG. Coordinate Regulation of Complex T Cell Populations Responding to Bacterial Infection. Immunity. 1998;8(3): 353–362. 10.1016/S1074-7613(00)80540-3 9529152

[17] Kamm C, Skoberne M, Geginat G. CD8 T cell immunome analysis of Listeria monocytogenes. FEMS Immunol Med Microbiol. 2003;35(3): 235–242. 12648842 10.1016/S0928-8244(02)00450-9

[18] Kipp M, van der Star B, Vogel DY, Puentes F, van der Valk P, Baker D, et al Experimental in vivo and in vitro models of multiple sclerosis: EAE and beyond. Mult Scler Relat Disord. 2012;1(1): 15–28. 10.1016/j.msard.2011.09.002 25876447

[19] Rangachari M, Kuchroo VK. Using EAE to better understand principles of immune function and autoimmune pathology. J Autoimmun. 2013;45: 31–39. 10.1016/j.jaut.2013.06.008 23849779 PMC3963137

[20] Procaccini C, De Rosa V, Pucino V, Formisano L, Matarese G. Animal models of Multiple Sclerosis. Eur J Pharmacol. 2015;759:182–91. 10.1016/j.ejphar.2015.03.042 25823807 PMC7094661

[21] Peterson LK, Fujinami RS. Inflammation, demyelination, neurodegeneration and neuroprotection in the pathogenesis of multiple sclerosis. J Neuroimmunol. 2007;184(1–2): 37–44. 10.1016/j.jneuroim.2006.11.015 17196667 PMC1933528

[22] Lassmann H, van Horssen J. The molecular basis of neurodegeneration in multiple sclerosis. FEBS Lett. 2011;585(23): 3715–3723. 10.1016/j.febslet.2011.08.004 21854776

[23] Fletcher JM, Lalor SJ, Sweeney CM, Tubridy N, Mills KH. T cells in multiple sclerosis and experimental autoimmune encephalomyelitis. Clin Exp Immunol. 2010;162(1): 1–11. 10.1111/j.1365-2249.2010.04143.x 20682002 PMC2990924

[24] Zepp J, Wu L, Li X. IL-17 receptor signaling and T helper 17-mediated autoimmune demyelinating disease. Trends Immunol. 2011;32(5): 232–239. 10.1016/j.it.2011.02.007 21493143 PMC3329781

[25] Xu S, Huo J, Tan JE, Lam KP. Cbp deficiency alters Csk localization in lipid rafts but does not affect T-cell development. Mol Cell Biol. 2005;25(19): 8486–8495. 16166631 10.1128/MCB.25.19.8486-8495.2005 PMC1265734

[26] Dobenecker MW, Schmedt C, Okada M, Tarakhovsky A. The ubiquitously expressed Csk adaptor protein Cbp is dispensable for embryogenesis and T-cell development and function. Mol Cell Biol. 2005;25(23): 10533–10542. 16287865 10.1128/MCB.25.23.10533-10542.2005 PMC1291250

[27] Hrdinka M, Horejsi V. PAG—a multipurpose transmembrane adaptor protein. Oncogene. 2014;33(41): 4881–4892. 10.1038/onc.2013.485 24213579

[28] Kravchick DO, Karpova A, Hrdinka M, Lopez-Rojas J, Iacobas S, Carbonell AU, Iacobas DA, Kreutz MR, Jordan BA. Synaptonuclear messenger PRR7 inhibits c-Jun ubiquitination and regulates NMDA-mediated excitotoxicity. EMBO J. 2016 7 25 pii: e201593070. 10.15252/embj.201593070 PMC5007554 27458189

[29] Gregoire C, Simova S, Wang Y, Sansoni A, Richelme S, Schmidt-Giese A, et al Deletion of the LIME adaptor protein minimally affects T and B cell development and function. Eur J Immunol. 2007;37(11): 3259–3269. 10.1002/eji.200737563 17918199

[30] Kolsch U, Arndt B, Reinhold D, Lindquist JA, Juling N, Kliche S, et al Normal T-cell development and immune functions in TRIM-deficient mice. Mol Cell Biol. 2006;26(9): 3639–3648. 16612002 10.1128/MCB.26.9.3639-3648.2006 PMC1447406

[31] Simeoni L, Posevitz V, Kolsch U, Meinert I, Bruyns E, Pfeffer K, et al The transmembrane adapter protein SIT regulates thymic development and peripheral T-cell functions. Mol Cell Biol. 2005;25(17): 7557–7568. 16107703 10.1128/MCB.25.17.7557-7568.2005 PMC1190311

[32] Liu Y, Zhang W. Identification of a new transmembrane adaptor protein that constitutively binds Grb2 in B cells. J Leukoc Biol. 2008;84(3): 842–851. 10.1189/jlb.0208087 18559951 PMC2516900

[33] Zhu M, Granillo O, Wen R, Yang K, Dai X, Wang D, et al Negative regulation of lymphocyte activation by the adaptor protein LAX. J Immunol. 2005;174(9): 5612–5619. 15843560 10.4049/jimmunol.174.9.5612

[34] Koelsch U, Schraven B, Simeoni L. SIT and TRIM determine T cell fate in the thymus. J Immunol. 2008;181(9): 5930–5939. 181/9/5930. 18941181 10.4049/jimmunol.181.9.5930

[35] Zhu M, Liu Y, Koonpaew S, Granillo O, Zhang W. Positive and negative regulation of FcepsilonRI-mediated signaling by the adaptor protein LAB/NTAL. J Exp Med. 2004;200(8):991–1000. 15477350 10.1084/jem.20041223 PMC2211849

[36] Fleming KK, Bovaird JA, Mosier MC, Emerson MR, LeVine SM, Marquis JG. Statistical analysis of data from studies on experimental autoimmune encephalomyelitis. J Neuroimmunol. 2005;170(1–2): 71–84. 10.1016/j.jneuroim.2005.08.020 16198426

