# Supplementary figures and images for: Normal Development and Function of T Cells in Proline Rich 7 (Prr7) Deficient Mice
Source: PLoS One. 2016 Sep 22;11(9):e0162863. doi: 10.1371/journal.pone.0162863 (PMC5033326; doi:10.1371/journal.pone.0162863)

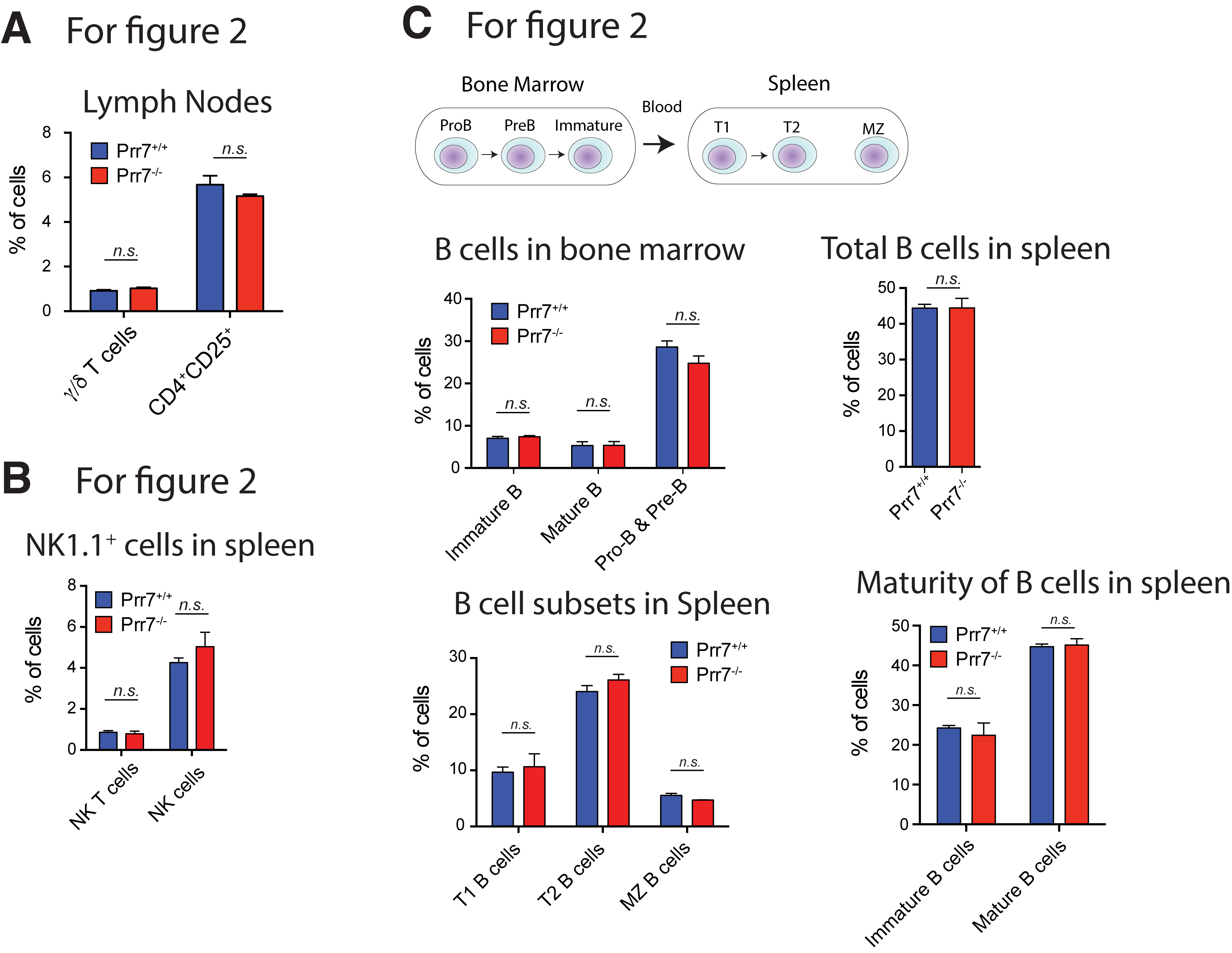

Supplement: S1 Fig — (A) Flow cytometry analysis of γ/δ T cell and enriched in regulatory T cells (CD4+CD25+) subpopulations in the lymph nodes. (B) Flow cytometry analysis of NK (NK1.1+TCRβ-) and NK T (NK1.1+TCRβ+) cells in the spleen stained with anti-NK1.1 and anti-TCRβ. (C) No gross defects were found in B cell development in Prr7 deficient animal. The upper schema depicts the main developmental stages of B cells in the bone marrow and in the spleen. MZ, marginal zone B cells. The middle and lower panels are the results of flow cytometry analysis of B cell developmental stages in the bone marrow and in the spleen of Prr7-/- mice and control Prr7+/+ mice defined as follow: Immature B cells (B220loIgM+), Mature B cells (B220hiIgM+), Pro & Pre B cells (B220+IgM-), T1 B cells (CD23+CD21+IgM+), T2 B cells (CD23+CD21+IgM+), MZ B cells (CD23-CD21+B220+), Immature B cells (IgMhiIgDlo), Mature B cells (IgMloIgDhi). Data in (A, B, C) represent the mean + SEM of at least three animals per group. n.s., not significant. (TIF) [file pone.0162863.s001.tif]

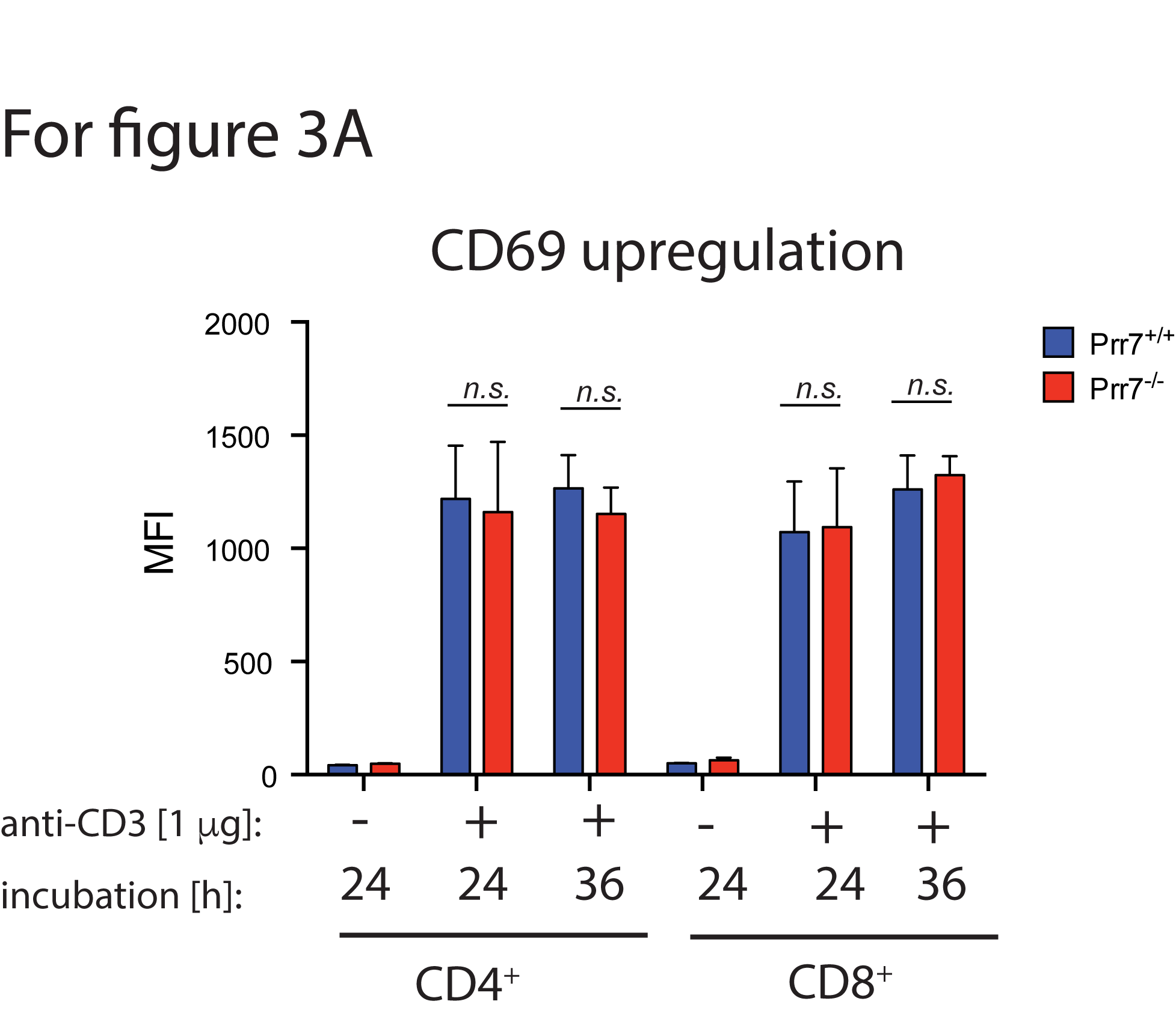

Supplement: S2 Fig — Data in represent the mean + SEM of minimum three animals per group. n.s., not significant. (TIF) [file pone.0162863.s002.tif]

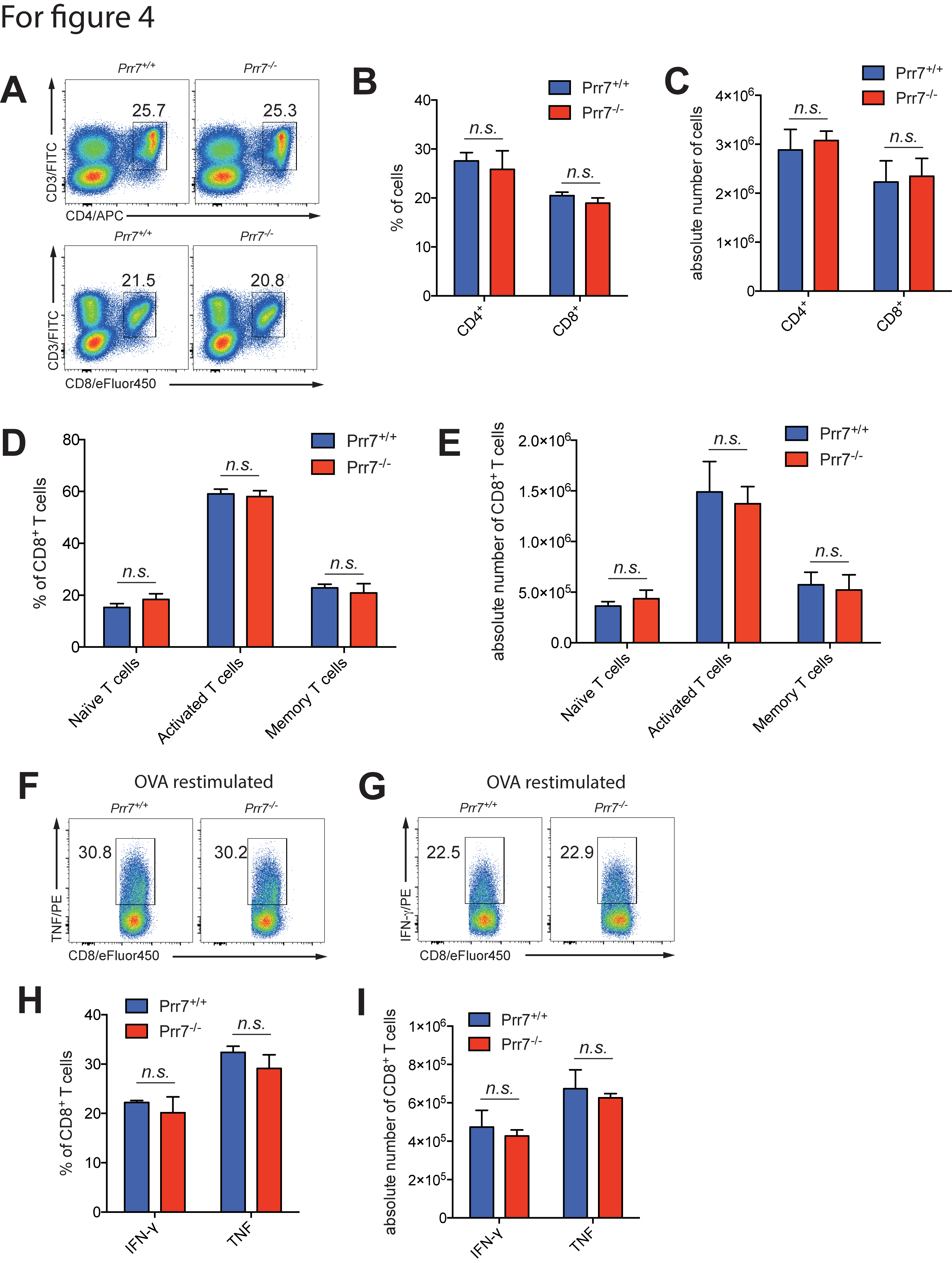

Supplement: S3 Fig — (A) Representative dot plot of CD4+ and CD8+ T cells in liver of Lm ova-infected mice. (B) Frequency of CD4+ and CD8+ T cells in liver of infected mice. (C) Absolute number of CD4+ and CD8+ T cells in liver of infected mice. (D) Frequency of CD8+ naive (CD62L+CD44-), activated (CD62L-CD44+) and memory (CD62L+CD44+) T cells in liver of infected mice. (E) Absolute number of CD8+ naive, activated and memory T cells in liver of infected mice. (F-I) Hepatic leukocytes of infected mice were restimulated with Ova257-264-peptide (SIINFEKL, 10−8 M) for 12 h in the presence of Brefeldin A. (F) Dot plot of TNF-producing CD8+ T cells. (G) Dot plot of IFN-γ producing CD8+ T cells. (H) Frequency of IFN-γ and TNF-producing CD8+ T cells in spleen of infected mice. (I) Absolute number of IFN-γ and TNF-producing CD8+ T cells in spleen of infected mice. Data are represented as mean + SEM of 3–4 mice per group. n.s. not significant. (TIF) [file pone.0162863.s003.tif]
